# Natural compounds modulating redox metabolism and Inflammation: New insights in skin cancer prevention and therapy

**DOI:** 10.1016/j.redox.2025.103913

**Published:** 2025-11-01

**Authors:** Sinemyiz Atalay Ekiner, Agnieszka Gęgotek, Elżbieta Skrzydlewska

**Affiliations:** Department of Analytical Chemistry, Medical University of Bialystok, Mickiewicza 2D, Białystok, 15-222, Poland

**Keywords:** Skin cancer, UV exposure, Catechins, Procyanidin C1, Piperitoside, Mulberrofurans

## Abstract

UV radiation penetrating human skin causes DNA damages, including specific mutations, redox imbalances, oxidative damage to macromolecules, and consequently changes in intracellular signaling pathways, particularly those involved in inflammation, cell differentiation, and survival. These metabolic changes may ultimately contribute to the development of skin cancers, including melanoma, the most lethal form, as well as other types of skin cancer. Although the link between climate change – a complex, multifactorial process that raises growing concerns about increased skin exposure to ultraviolet (UV) radiation – and the development of skin cancers has not yet been fully explored, the issue is gaining attention. Intensive and multifaceted research is underway to identify natural compounds with anticancer, preventive, and regenerative properties. This review highlights promising natural compounds (catechins, procyanidin C1, piperitoside, and mulberrofurans), some of which are already known to have effects on skin cancers. Their common feature is the ability to regulate redox signaling and inflammation, which play key roles in both the prevention and treatment of skin cancer.

## Highlights

• Natural compounds that modify redox metabolism may help fight skin cancer.
• Catechins (EGCG, GCG, CG) demonstrate inhibitory action on skin tumor growth.
• Procyanidin C1 demonstrates anti-fibrotic and anti-proliferative actions.
• Piperitoside may modulate UV-induced immunosuppression by altering skin microbiota.
• Mulberrofurans can modulate tumor cell survival by interfering protein aggregation.

## Graphical abstract

**Figure:**
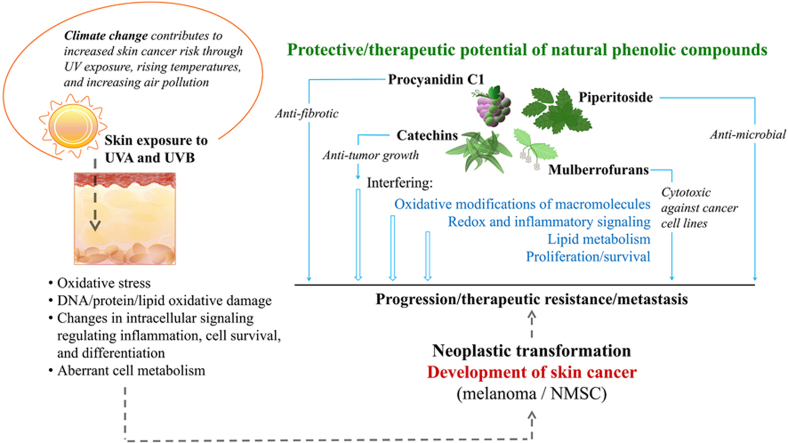

## 1 Introduction

Climate change is a rapidly growing problem that requires a multidisciplinary analysis of various components and their environmental effects [1]. These effects include increased human exposure to UV radiation in sunlight [1], which predominantly consists of UVA (320–400 nm) and UVB (290–320 nm), as well as UVC (200–290 nm); however, UVC is absorbed by atmospheric ozone [2]. UVA penetrates the dermal stroma, while UVB is primarily absorbed by the epidermis [3]. Therefore, human skin that contributes to maintain the body's homeostasis and serves as a physical barrier between the environment and human organism, is particularly susceptible to these consequences [4].

**Fig. 1:**
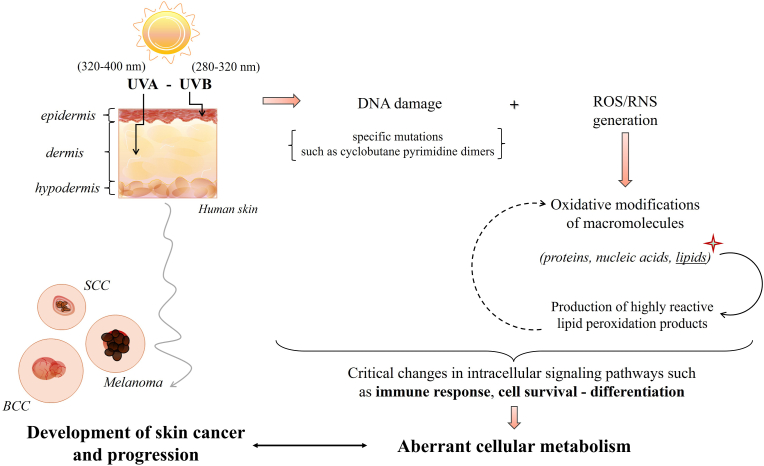
Brief summary of skin cancer development and progression through the effect of UV-caused redox imbalance. [**BCC**, basal cell carcinoma; **SCC**, squamous cell carcinoma; **ROS**, reactive oxygen species; **RNS**, reactive nitrogen species; **UVA**, ultraviolet A; **UVB**, ultraviolet B].

UV irradiation causes various types of DNA damages and specific mutations, such as cyclobutane pyrimidine dimers, which are particularly associated with melanoma, notably induced by sunlight (especially UVB radiation) [5]. It is estimated that UVA may contribute 10–20 %, while UVB accounts for 80–90 % of the sunlight dose responsible for inducing skin carcinogenesis [5]. In addition to their direct mutative effects, they also trigger reactive oxygen and nitrogen species (ROS and RNS) generation, leading to downstream oxidative damage to macromolecules (DNA, proteins, and lipids) and ultimately resulting in aberrant cellular metabolism [6]. Among other effects, by provoking ROS and RNS generation, UV radiation also induces the formation of highly reactive lipid peroxidation products [7]. Consequently, both ROS/RNS and lipid peroxidation products can critically alter cell metabolism by modifying the structure of macromolecules, thereby affecting key intracellular signaling pathways that regulate inflammation, cell survival, and differentiation [8]. Ultimately, UV radiation may disrupt cellular homeostasis and metabolism [9]. This situation may lead to various health problems, including local ones (sunburn, inflammation) and systemic ones (photoimmunosuppression and photoaging) [9]. Even, metabolic changes at the skin level can ultimately lead to the development of skin cancers (Fig. 1), including the most lethal form of skin cancer - melanoma as well as non-melanoma skin cancer (NMSC), such as squamous cell carcinoma (SCC) and basal cell carcinoma (BCC) [2,9,10]. Several epidemiological studies indicate a rising incidence of both NMSC and melanoma over the past several decades [11]. NMSC is the most common type of skin cancer, accounting for about 1/3 of all diagnosed malignancies [12]. Melanoma, although it accounts for less than 10 % of skin cancer cases, is characterized by the highest aggressiveness and high mortality [13].

Despite the lack of a systematic analysis of the complexities in the relationship between climate change and skin cancer, it is strongly suggested that climate change can contribute to increased skin cancer risk through UV exposure, rising temperatures, and increasing air pollution [14]. Therefore, various natural compounds with anticancer activity are being sought and tested. By targeting redox signaling, inflammation, and cell survival, these compounds may prevent the harmful influence of environmental stressors, including UV radiation, on skin cell metabolism [15]. Due to their potential for fewer adverse effects compared to most non-selective chemotherapeutic drugs [16] and their rich content of bioactive compounds, natural products and their structural analogues have made significant contributions to pharmacotherapy, particularly in cancer [17]. Despite limitations such as technical barriers to screening, isolation, characterization, and optimization, their advantages in large-scale production and culturing [17] make them strong candidates for future pharmacotherapeutic applications.

Many of the natural compounds, including polyphenols, flavonoids, vitamins, alkaloids, terpenoids, cannabinoids, carotenoids, and ceramides from natural sources possess anti-cancer properties, and their potential in the prevention and treatment of skin cancer is increasingly being investigated [15]. Although they belong to different chemical classes, they share the common ability to interfere with intracellular redox and inflammatory signaling, as well as related cellular processes such as cell death pathways [15]. This is highly important, given that ROS signaling and the immune-inflammatory response, which interact with each other [18], are key contributors to skin carcinogenesis [19], as well as to carcinogenesis in general [20]. Owing to their redox-active chemical nature, phenolic compounds represent an important class of natural molecules, capable of directly influencing cellular redox status and associated signaling pathways including inflammation [21].

Although research in this field is rapidly evolving, further studies are still needed to identify novel compounds and to elucidate their underlying mechanisms of action in the complex and dynamic cellular metabolism of skin cancer. Therefore, this review highlights both previously and currently studied natural phenolic compounds, as well as those identified as having potential to regulate redox signaling and/or related inflammatory pathways, which may help prevent the development of various skin cancers and demonstrate pharmacotherapeutic potential.

## 2 A methodological framework for selecting natural phenolic compounds

This systematic study highlighted natural phenolic compounds that demonstrate potential in skin cancer therapy. The analysis covered literature data from 2005 to 2025. Considering the regulatory role of redox metabolism in the development of skin cancers, phenolic (natural) compounds were selected based on two criteria: (**1**) compounds that modulate intracellular redox balance and/or inflammatory signaling and induce metabolic changes associated with them; (**2**) compounds that demonstrate protective/therapeutic effects (*in vitro/in vivo/in clinical trials*) primarily in skin cancers, including BCC, SCC, or melanoma. Due to limited data on skin cancer, information from other cancer types was also included, particularly regarding the regulatory role of these compounds in cell molecular biology. However, it was noted that biological differences specific to individual tumors may influence the results. In this review, we also emphasized the importance of examining compounds that have previously been proven effective in treating various types of cancer as potential candidates for skin cancer therapy. At the same time, we emphasize that a thorough analysis of variability is essential to clearly assess their efficacy in the tissue-specific context of carcinogenesis, especially in different skin cancer subtypes.

## 3 Skin cancer: the interplay of oxidative stress and lipid metabolism

### 3.1 Molecular insights into skin cancer

Skin, presents a series of layered interfaces such as: The outermost epidermis layer of stratified squamous epithelium that spontaneously regenerates, a basement membrane zone and a fibrous neurovascular dermis layer, which is resting on a hypodermis or subcutaneous fat [22]. Epidermis mainly consists of keratinocytes, but also non-epithelial cells, including antigen-presenting dendritic Langerhans cells as well as melanocytes and Merkel cells [22]. Dermis layer allows an absorbing face against mechanical forces with collagen fibers and its major cell type, fibroblasts, which produce extracellular structural proteins, glycosaminoglycans, collagen and elastin fibers [23].

This multilayered structure, along with the developmental characteristics of skin ontogeny, gives rise to a wide molecular network involved in the maintenance, regeneration, and differentiation of skin tissue. Disruption of this complex network may drive pathologies, including carcinogenesis in the skin, a critical interface between the body and environmental stressors. There are two major types of skin cancer: melanoma, which arises from melanocytes dysfunction, and NMSC, which arise from abnormalities in epidermal-derived cells and is mainly divided into two subtypes: SCC and BCC [24]. BCC develops from the outermost layer of the epidermis and closely resembles basal cells, whereas SCC originates from keratinocytes located in both squamous and non-squamous epithelial tissues [25]. NMSC is the most common type of cancer while melanoma is the utmost severe and deadliest form of skin cancer [26].

Large-scale "omics" studies, including genomics, proteomics and metabolomics, have improved the understanding of the molecular landscape of skin cancer – a complex and multifactorial disease – while also providing better opportunities for diagnosis and classification, similar to various other cancer types [27]. The genomic profile of skin cancer demonstrates considerable complexity. In cutaneous melanoma, the most frequently genetic subtypes are *mutant serine/threonine-protein kinase B-raf* (BRAF) and *GTPase NRas* (NRAS), *neurofibromin 1* (NF1) and *triple wild-type* [28]*.* Among others, mitogen-activated protein kinase (MAPK)-activating mutations are also highlighted as critical in the pathogenesis of melanoma [28]*.* In SCC, mutations commonly occur in the NOTCH and p53 pathways, along with alterations in genes associated with the Hippo signaling pathway [Ras(GTPase)/MAPK/phosphatidylinositol 3-kinase (PI3K)] [29]. However upregulation of N-Myc (bHLH transcription factor) and Hippo-YAP pathway target genes is indicated in BCC tumorigenesis [30]. Also recent findings suggests uncover solute carrier family 45 member 2 (SLC45A2), regulator of chromosome condensation 2 (RCC2), and CLPTM1L (CLPTM1 regulator of GABA type A receptor forward trafficking like) as genetic risk factors for BCC in Hispanic/Latinos [31].

A study analyzing the proteomes of melanoma subtypes highlights 140 proteins that overlap with cancer hallmarks, tumor suppressors, and regulators of metabolism and the cell cycle [32]. This study underlines the aberrant activation of the phosphoinositide 3-kinase-protein kinase B - mammalian target of rapamycin (PI3K-Akt-mTOR) and inactivation of the Hippo-YAP pathway, acting as a tumor suppressor [33] in benign-to-malignant transition in melanomas [32]. Another proteomic study shows significant increase of CEACAM1 (carcinoembryonic antigen-related cell adhesion molecule 1), MC1R (melanocortin 1 receptor), AKT1 (protein kinase B) and MMP3-9 (matrix metalloproteinases 3 and 9) and decrease in CDKN2A (cyclin-dependent kinase inhibitor 2A), SDC1 and SDC4 (syndecan 1 and 4) protein levels in distant organ metastatic melanomas [34]. Elevated levels of sodium-hydrogen antiporter 3 regulator 1 (SLC9A3R1), B-lymphocyte surface antigen B1 (CD20), and growth factor receptor bound protein 2 (GRB2), along with reduced levels of cystatin-M (CST6), serpin family B member 5 (SERPINB5), and arginase-1 (ARG1), are found to be associated with regional lymph node metastasis in melanomas [34].

However the study conducted on e-biopsy samples from cutaneous SCC and BCC lesions revealed overexpression of cornulin (CRNN), estrogen sulfotransferase (SULT1E1), and inositol-tetrakisphosphate 1-kinase (ITPK1) in BCC, compared SCC [35]. Moreover, in the case of BCC, MMP1 due to its role in increasing cell motility and nevus invasion is also suggested as a new marker for targeted therapy of this cancer [36]. Significant increase in transforming growth factor beta (TGF-β), Smad2, cathepsin-K, progerin and MMP1, 3, 8 and 9 expressions is highlighted in skin biopsies with diagnosed BCC [37].

A study suggested pharmacological blockade of mitogen-activated protein kinase 2 (MAP2K2) activity for the treatment of recurrent human papillomavirus - driven head and neck SCC [38]. According to overexpression of epidermal growth factor receptor (EGFR), same study also reveals altered kinase signaling network involving MAP2K2 to regulate the aberrant changes in the recurrent cancer cells [38]. Moreover, mutations of cyclin D1 (CCND1) and RB transcriptional corepressor 1 (RB1), fibroblast growth factor receptor 1 (FGFR1) and phosphatidylinositol-4,5-bisphosphate 3-kinase catalytic subunit alpha (PIK3CA), tumor protein p63 (TP63), SRY-Box transcription factor 2 (SOX2), and neurogenic locus notch homolog protein 1 (NOTCH1) as well as lysine N-methyltransferase 2C and 2D (KMT2C and KMT2D) genes have been identified in SCC [39]. Also, diagnostic values of protein kinase C epsilon type (PRKCE) for vaginal and cervical SCCs, carboxypeptidase X, M14 family member 2 (CPXM2) for thymus SCCs, and solute carrier family 27 member 1 (SLC27A1) for pancreatic and gallbladder SCCs have been remarked [39].

### 3.2 Redox regulation in skin cancer

#### 3.2.1 Key players: redox sensitive proteins

The literature emphasizes the critical role of redox signaling in the oncogenesis of skin cancer [19]. Modulation of redox-sensitive proteins (peroxiredoxin 3/5, PRX3/PRX5, selenoenzyme 2, TR2, glutathione reductase, GR, and NADPH oxidase 4, NOX4) is suggested as a target for cancer therapy due to their metabolic role in various stages of melanoma, from initiation to metastasis and resistance [40]. Abnormal nuclear factor erythroid 2-related factor 2 expression (Nrf2, the master regulator of the antioxidant defense system) is associated with melanoma incidence and is co-opted during cancer progression, with its levels varying by cancer stage [41]. In addition, maternal embryonic leucine zipper kinase (MELK)-mediated melanoma growth by activating nuclear factor kappa B (NF-κB) pathway via sequestosome 1 (SQSTM1/p62) has been shown in BRAF-mutant melanoma cell lines [42].

Therefore, it has been suggested that combining targeted “oxidative stress pathway therapies” with other treatment modalities, such as chemotherapy or immunotherapy, may enhance treatment response, particularly in aggressive or resistant disease [43]. Moreover, one recent clinical study highlights increased levels of oxidative damage markers in NMSC patients compared to the control group [44]. NMSC patients shows lower catalase activity and glutathione (GSH) level compared to non-cancer patients [44]. The same study indicates that higher vitamin D levels correlate with lower oxidative stress, showing a positive correlation with GSH levels and catalase activity in erythrocytes [44]. An important finding is that UV-induced oxidative protein modifications are involved in cancer initiation, including carbonylation defined as a critical factor promoting cancer development by increasing genomic instability and tumor progression [45].

#### 3.2.2 ROS generation and antioxidant defense

Considering metabolic changes, cancer cells are characterized by higher levels of ROS compared to the non-cancerous cells from which they originate [46]. Literature indicates this situation as a primary cause or an aggravating factor in the primary condition of oxidative stress in the development and/or prognosis of many skin diseases, including melanoma [47]. In skin cancer, oxidative stress can shape the pathogenesis in diverse ways. UV-mediated impairment of antioxidant defense has been implicated in the multistep carcinogenesis of NMSC, whereas increased oxidative stress in melanoma cells, which can damage surrounding tissue, may promote metastatic progression [48]. Due to oxidative modifications of nucleic acids, proteins, and lipids, as well as related alterations of intracellular signaling – by impairing the intracellular antioxidant defense – UV radiation contributes to melanoma pathogenesis and even immunosuppression in exposed skin tissue [49]. This can lead to immune surveillance evasion and further disease progression [49].

Looking more closely, melanocytes are mainly indicated, as they are more exposed to oxidative stress than other cells due to melanin production [47]. Regarding melanoma, in addition to role of oxidative stress in malignancy development, its involvement in chemoresistance makes oxidative stress a target for therapeutic applications [47]. To mitigate the harmful effects of increased intracellular ROS and RNS levels, melanoma cells employ multiple redox modulators that enhance their antioxidant defenses, engaging metabolic pathways including the pentose phosphate pathway, lipogenesis, and mitochondrial function [50]. In addition, an oxidative stress-related lncRNA (long non-coding RNAs) signature has been demonstrated as associated with melanoma progression [51]. Literature indicates that antioxidant enzymes such as catalase, glutathione peroxidase, and superoxide dismutases (SOD1 and SOD2) may mitigate tumor cell proliferation, by regulating ROS generation and T-cell activation [52]. However, antioxidants have also been also found to be linked to melanoma progression and metastasis [53]. Particularly, elevated GSH levels have been observed in melanoma cells, enhancing their survival under oxidative stress [50]. This highlights the complex role of redox homeostasis in melanoma genesis and progression [53], as well as the dose-dependent effects observed in clinical studies of natural compounds with antioxidant activity.

Excessive ROS and RNS generation has been shown to drive the development and progression of SCC, and BCC via both genotoxic and non-genotoxic pathways. Increased SOD and GSH levels have been reported in BCC patients [54]. Moreover, regarding the interplay between skin microbiota, oxidative stress, and carcinogenesis, recent studies also highlight the potential role of dysbiosis-mediated oxidative stress (through pro-oxidant activities and/or metabolites of some microbiota species) in onset and progression of skin cancer [54]. Thus, oxidative stress and antioxidant defense mechanisms constitute a critical and multifaceted target for therapeutic strategies against skin cancers.

#### 3.2.3 Lipid metabolism: contribution of reactive lipids

Lipids which play a key role in cell signaling and physiology, particularly ω-3 and ω-6 polyunsaturated fatty acids (PUFAs), are exceptionally susceptible to oxidative modifications due to their chemical moieties [55]. However, α,β polyunsaturated aldehydes, which are products of lipid metabolism, exhibit electrophilic properties and interact with proteins, nucleic acids, modifying their structure and intracellular signaling [56]. This, in turn, influences autophagy, cell proliferation, transcriptional control, and apoptosis [56]. Thus, the changes in lipid profile and lipid metabolism open a crucial opportunity for targeted therapies.

Lipid metabolism was likely more active and plays key roles in squamous cell differentiation in metaplastic SCC [39]. Changes in the expression of key lipid metabolism proteins have been identified in head and neck SCCs: low-density lipoprotein receptors (LDLR), platelet glycoprotein 4 (CD36), the lectin-like oxidized LDL receptor-1 (LOX-1), fatty acid binding proteins on the plasma membrane (FABPs), ATP-citric acid lyase (ACLY), acetyl-CoA acetyltransferase, mitochondrial (ACAT1), HMG-CoA reductase (HMGCR), fatty acid synthase (FASN), fatty triglyceride lipase (ATGL), carnitine palmitoyltransferase 1 (CPT1), stearoyl-CoA desaturase (SCD), acetyl-CoA carboxylases (ACCs) [57]. A recent study showing the regulatory effect PUFAs (including arachidonic acid and eicosapentaenoic acid) on the progression of BCC by indicating correlation between increased activity of PUFA desaturase and the risk of developing BCC [58,59]. Additionally, numerous research studies indicate that alterations in the lipid metabolic network contribute to cell growth and the metastasis of melanoma cells [60]. Thus, further analysis of the PUFAs synthesis pathway, including the activity of FASN, ACLY, and ACCs [61], and its effect on cell signaling needs to be evaluated.

The regulatory role of the cyclooxygenase-2/prostaglandin E2 (COX-2/PGE2) pathway in mediating immune escape and promoting tumor progression in melanoma has been well-documented [62]. Inhibition of DGAT1 (diacylglycerol O-acyltransferase 1, an enzyme responsible for triacylglycerol synthesis) activity has also been found to cause excessive fatty acid oxidation, ROS generation and associated Nrf2 activation, which may inhibit tumor growth by inducing intolerable oxidative stress [63]. Moreover, an interesting study profiling the proteome of DGAT1 lipid droplets revealed that these droplets enhance the acquisition of a more diverse transcriptional identity in melanoma cells [64]. These results underlined the critical role of the lipid droplet proteome, which includes a diverse range of metabolic, signaling, transport and membrane organizing proteins. In addition, the same study indicated the importance of increased fatty acid uptake, along with a higher number of lipid droplets in the melanocytic state, which is suggested to be driven by fatty acid oxidative metabolism.

Therefore, the indicated data on metabolic changes accompanying the development of skin cancers, including the interaction of oxidative stress with inflammation which is accompanied by modifications in lipid and protein structure and metabolism confirm that, in the case of skin cancer, it is worth paying attention to natural compounds with redox regulation potential, which will be discussed in the following sections of this article.

#### 3.2.4 The role of energy metabolism

**Fig. 2:**
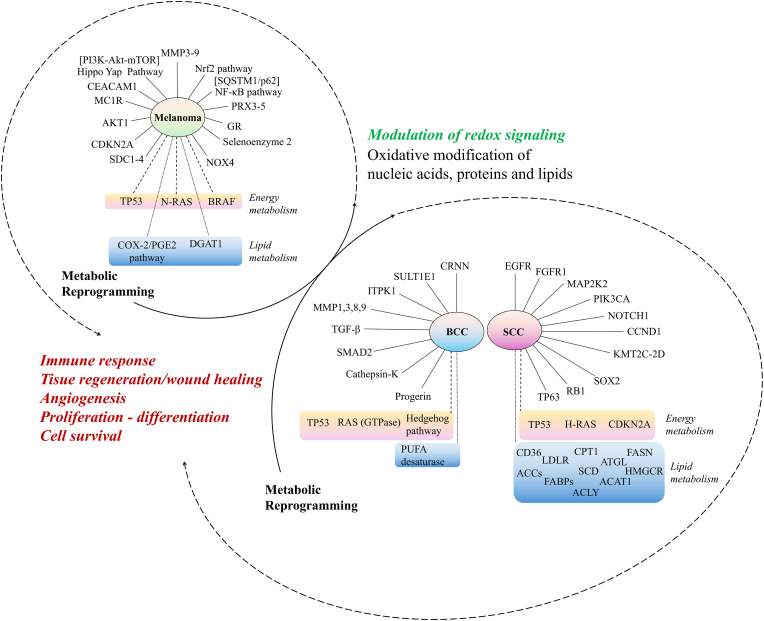
Prominent proteomic profile under the influence of the interaction between intracellular redox signaling and metabolic reprogramming, which play key roles in inflammation, differentiation and survival dynamics in skin cancer. [**ACAT1**, acetyl-CoA acetyltransferase, mitochondrial; **ACCs**, acetyl-CoA carboxylases; **ACLY**, ATP-citric acid lyase; **AKT1/AKT**, protein kinase B; **ATGL**, fatty triglyceride lipase; **BCC**, basal cell carcinoma; **BRAF**, serine/threonine-protein kinase B-raf; **CCND1**, cyclin D1; **CD36**, platelet glycoprotein 4; **CDKN2A**, cyclin-dependent kinase inhibitor 2A; **CEACAM1**, carcinoembryonic antigen-related cell adhesion molecule 1; **COX-2**, cyclooxygenase-2; **CPT1**, carnitine palmitoyltransferase 1; **CRNN**, cornulin; **DGAT1**, diacylglycerol O-acyltransferase 1; **EGFR**, epidermal growth factor receptor; **FABPs**, fatty acid binding proteins on the plasma membrane; **FASN**, fatty acid synthase; **FGFR1**, fibroblast growth factor receptor 1; **GR**, glutathione reductase; **HMGCR**, HMG-CoA reductase; **H-RAS**, GTPase HRas; **ITPK1**, inositol-tetrakisphosphate 1-kinase; **KMT2C-2D**, lysine N-methyltransferase 2C and 2D; **LDLR**, low-density lipoprotein receptors; **MAP2K2**, mitogen-activated protein kinase 2; **MC1R**, melanocortin 1 receptor; **MMP1,3,8,9**, matrix metalloproteinases 1,3,8 and 9; **mTOR**, mammalian target of rapamycin; **NF-κB**, nuclear factor kappa B; **NOTCH1**, neurogenic locus notch homolog protein 1; **NOX4**, NADPH oxidase 4; **N-RAS**, neuroblastoma RAS proto-oncogene, GTPase; **Nrf2**, nuclear factor erythroid 2-related factor 2; **PGE2**, prostaglandin E2; **PI3K**, phosphatidylinositol 3-kinase; **PIK3CA**, phosphatidylinositol-4,5-bisphosphate 3-kinase catalytic subunit alpha; **PRX3-5**, peroxiredoxin 3-5; **PUFA**, polyunsaturated fatty acids; **RAS**, GTPase; **RB1**, RB transcriptional corepressor 1; **SCC**, squamous cell carcinoma; **SCD**, stearoyl-CoA desaturase; **SDC1-4**, syndecan 1–4; **SOX2**, SRY-Box transcription factor 2; **SQSTM1/p62**, sequestosome 1; **SULT1E1**, estrogen sulfotransferase; **TGF-β**, transforming growth factor beta; **TP53**, tumor protein p53; **TP63**, tumor protein p63].

On the other hand, since the reprogramming of cellular metabolism is considered as a hallmark of cancer, the significance of energy metabolism comes to the forefront in the initiation and progression of skin cancer [65] (Fig. 2). Mutations in the Hedgehog pathway, tumor protein p53 (TP53), the RAS (GTPase) gene family in cutaneous BCC and CDKN2A (Cyclin-dependent kinase inhibitor 2A), TP53, H-RAS in cutaneous SCC as well as TP53, NRAS and BRAF proto-oncogenes in melanoma draw attention in the context of altered energy metabolism [65,66]. Furthermore, molecular traces of metabolic reprogramming of carcinogenesis appear at the level of redox balance, mitochondrial respiration and glycolysis. Regarding chronic inflammation and neutrophil extracellular traps (NETs), it is important to evaluate cellular response to target therapies in the concept of endogenous mitochondrial ROS generation and alteration of glucose metabolism [67]. Relying on oncogenic alterations redirected glucose away from the mitochondria, cancer cells shows an extensive dependence on glutaminolysis to support swift ATP generation and biosynthesis while simultaneously regulating ROS generation [46]. Thus, identifying the intricate molecular crosstalk between mitochondrial metabolism, oxidative stress, inflammation, apoptosis and the molecular action of natural compounds interfering with these pathways is crucial for future therapies.

## 4 Promising natural phenolic compounds in the prevention/treatment of skin cancer; new aspects

Advancements in analytical tools, genome mining, engineering strategies, and high-capacity culturing have positioned natural compound-based drug discovery as a major opportunity in pharmacotherapy, particularly for treating cancer and infectious diseases [17]. Owing to hydroxyl substitutions on their aromatic rings and the presence of conjugated double bonds, phenolic compounds have garnered significant attention for their antioxidant and anticancer activities, particularly in combination approaches for chemoprevention in cancer treatment [68]. Phenolic acids, have been identified as promising anticancer candidates owing to their shown capacity to modulate oxidative stress, apoptosis, and inflammation [69]. Phenolics are reported to possess anti-angiogenic effects, primarily through the suppression of invasion and angiogenesis [70]. However, condensed tannins, such as proanthocyanidins, have been reported with strong free-radical scavenging and antitumor activity [71]. In this section, we highlight promising natural phenolic compounds for their potential protective and therapeutic roles in skin cancers.

### 4.1 Catechins from *Camellia sinensis*

*Camellia sinensis*, is highlighted with high levels of phenolic compounds known as catechins (belonging to the flavonoids) that present strong antioxidant properties [72]. Catechins also present significant anti-inflammatory properties through the modulation of activity of Nrf2, NF-κB, MAPKs, signal transducer and the activator of transcription 1/3 (STAT1/3) [73]. This makes them particularly important for pathologies accompanied by oxidative stress and chronic inflammation.

**Fig. 3:**
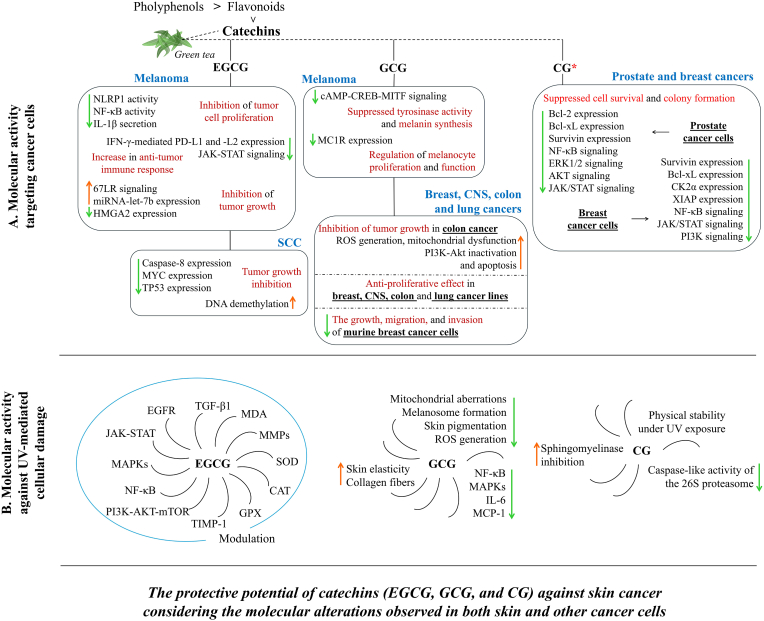
Catechins (EGCG, GCG, and CG) modulate protein expression, intracellular redox status, and molecular signaling pathways in both skin and other cancer cells. They also influence intracellular dynamics in response to UV-induced cellular damage, including redox imbalance, macromolecular disruption, and structural damage. These molecular; changes, observed in both skin and other cancer cells, highlight the therapeutic potential of the mentioned catechins against melanoma and non-melanoma skin cancers (NMSC). The mentioned catechins alter intracellular molecular signaling by modifying protein expression in cells derived from melanoma/SCC (segment **A**), and other cancer types, including breast, central nervous system (CNS), colon, pancreatic, and lung cancers (segment **B**). “∗” indicates that the effectiveness of this catechin against skin cancer has not yet been determined, but it shows a high promising potential. Green arrows indicate a reduction in expression or suppression of related molecular signaling. Orange arrows a rise in expression or suppression of related molecular signaling. Orange arrows. Segment B summarizes the modulation of protein expression and associated molecular signaling by the indicated catechins. [**67LR**, 67-kDa laminin receptor; **Akt**, protein kinase B; **Bcl-2**, B-cell lymphoma 2; **Bcl-xL**, B-cell lymphoma-extra large; **cAMP**, cyclic adenosine monophosphate; **CAT**, catalase; **CG**, (−)-catechin-3-*O*-gallate; **CK2α**, casein kinase 2 subunit alpha; **CREB**, cAMP-responsive element-binding protein; **EGCG**, (−)-epigallocathechin-3-gallate; **EGFR**, epidermal growth factor receptor; **ERK1/2**, extracellular signal-regulated kinases 1/2; **GCG**, (−)-gallocatechin gallate; **GPX**, glutathione peroxidase; **HMGA2**, high mobility group A2; **IFN-γ**, interferon-gamma; **IL-1β**, interleukin-1 beta; **IL-6**, interleukin 6; **JAK**, the janus kinase; **MAPKs**, mitogen activated protein kinases; **MC1R**, melanocortin 1 receptor; **MCP-1**, monocyte chemoattractant protein-1. **MDA**, malondialdehyde; **miRNA-let-7b**, microRNA let-7b; **MITF**, microphthalmia-associated transcription factor; **MMPs**, matrix metalloproteinases; **mTOR**, mammalian target of rapamycin; **MYC**, MYC proto-oncogene, bHLH transcription factor; **NF-κB**, nuclear factor kappa B; **NLRP1**, NLR family pyrin domain containing 1; **PD-L1/-L2**, programmed death-1, ligand 1 or 2; **PI3K**, phosphatidylinositol 3-kinase; **SOD**, superoxide dismutase; **STAT**, signal transducer and activator of transcription; **TGF-β1**, transforming growth factor-beta1; **TIMP-1**, tissue inhibitor of metalloproteinase-1; **TP53**, tumor protein p53; **XIAP**, X-linked inhibitor of apoptosis protein].

Major catechin of green tea is (−)-epigallocathechin-3-gallate (EGCG). Indeed, its epimer (−)-gallocatechin gallate (GCG) has been reported to be even more stable and bioactive than EGCG, due to auto-oxidation and epimerization rate of EGCG contributing to its instability [74,75]. Another catechin, at relatively minor concentration, is (−)-catechin-3-*O*-gallate (CG). It is a gallate ester and a polyphenol [76]. This metabolite has been notably less studied than the others mentioned herein. On the other hand, EGCG's role in interfering with various molecular pathways (EGFR, JAK-STAT, MAPKs, NF-κB, PI3K-AKT-mTOR) was demonstrated, and its therapeutic potential, especially in cancer, has been highlighted [77]. Preclinical and clinical studies have emphasized its potential role in the modulating cell proliferation, inflammatory responses, apoptosis, and epigenetic alterations (Fig. 3).

#### 4.1.1 (−)-epigallocathechin-3-gallate (EGCG, PubChem CID: 65064)

A study on skin fibroblasts exposed to UVA radiation reveals that EGCG (25 μg/mL) can effectively reverse the UVA-induced inhibition of transforming growth factor-beta1 (TGF-β1) secretion and the levels of antioxidant enzymes – superoxide dismutase (SOD), catalase (CAT), and glutathione peroxidase (GPX) [78]. Another study also demonstrated the antioxidant activity of EGCG (12.5 and 25 μM) in HaCaT cells and its ability to inhibit radical-induced apoptosis by downregulating caspase-8 and -3, along with a reduction in melanin production and secretion (50 μM) in melanoma cells [79]. EGCG also counteracts the UVA-induced increase of G1-phase cells (cell cycle arrest in the G1 phase to prevent further DNA damage and reduce the risk of gene mutations), malondialdehyde (MDA) production, as well as the expression of matrix metalloproteinases (MMPs) and tissue inhibitor of metalloproteinase-1 (TIMP-1) [78]. Recent findings highlight the potential protective role of EGCG against skin photoaging by improving skin elasticity and hydration, reducing pigmentation, and reversing UVA-induced molecular damages as mentioned above [80].

In SSC-4 human oral squamous cell carcinoma, EGCG treatment (20, 50, 100, and 200 μM) caused tumor cell death through apoptosis and autophagy, dose-as well as time-dependently [81]. It reduced mitochondria transmembrane potential by down-regulating of caspase-8 (CASP8), MYC, and TP53 expression [81]. Also, DNA demethylating effect of EGCG and associated tumor growth inhibition potential have been demonstrated on the head and neck SCC [82].

EGCG (0.1 and 1 μM) has been shown to inhibit melanoma growth through a detailed mechanism involving the downregulation of inflammasome NLRP1 (NACHT-, LRR- and pyrin-domain-containing protein 1) and decreased NF-κB activity, IL-1β secretion and cell growth [83]. Another study demonstrated that EGCG enhances anti-tumor immune responses, as shown through both *in vitro* (10 μM) and *in vivo* (50 and 100 mg/kg) experiments [84]. EGCG treatment caused a significant reduction in interferon-gamma (IFN-γ)-induced PD-L1 and PD-L2 (programmed death-ligand 1 and 2) expression, as well as JAK-STAT signaling [84]. On the other hand, *in vivo* studies using C57BL/6 mice showed that EGCG-mediated tumor suppression occurred through the activation of CD8^+^ T cells [84]. Moreover, another study focusing on microRNAs (miRNAs) proposed a mechanism of EGCG (10 μM) action involving activation of 67-kDa laminin receptor (67LR) signaling, upregulation of miRNA let-7b, and downregulation of high-mobility group A2 (HMGA2) expression. These led to EGCG-induced inhibition of melanoma tumor growth [85].

The therapeutic potential of EGCG in skin cancer, including melanoma, has been highlighted by several preclinical studies. However, although ongoing research exists (focused primarily on breast cancer), clinical trials specifically focused on skin (particularly skin cancer) remain limited. Topical treatment with EGCG has been shown to protect the skin of nude mice against UVB-induced photoaging, which is observed by reduced antioxidant levels (SOD and GPX) and increased inflammatory response (tumor necrosis factor-α, TNF-α) [86]. A pilot *in vivo* study in a mouse SCC model showed that EGCG-enhanced cream (conventional cream containing methyl aminolevulinate 20 % + EGCG 3 %) significantly reduced lesions when used alongside photodynamic therapy [87]. A phase I study showed the safety of EGCG solution (from 660 to 2574 μmol/L) and beneficial effect in patients with radiation-induced dermatitis (grade III) receiving radiotherapy [88]. In another clinical study involving 16 healthy volunteers, oral administration of green tea extract (containing 40.3 % EGCG) and vitamin C counteracted skin inflammation caused by UVA/UVB irradiation, as evidenced by a reduction in the inflammatory mediator 12-hydroxyeicosatetraenoic acid (12-HETE) in skin biopsy and blister fluid samples [89]. However, levels of PGE2 remained unchanged [89]. Another trial (involving 50 healthy volunteers) highlighted that oral administration of green tea extract, containing high levels of EGCG and vitamin C (total daily dose of extracts: 1080 mg), reduced the degradation of skin elastin fibers caused by UV irradiation, helping maintain skin elasticity and supporting the structural integrity of fibulin-5 [90]. Although clinical studies are promising [91] more comprehensive *in vivo* and clinical investigations specifically targeting melanoma and NMSC are needed.

#### 4.1.2 (−)-Gallocatechin gallate (GCG, PubChem CID: 199472)

Another catechin, GCG, has been analyzed as a potential protective compound against skin photodamage. GCG treatment has been shown to reduce mitochondrial aberrations and melanosome formation in the UVB-irradiated skin of hairless mice [92]. Topically applied GCG, at low (25 mg/mL) and high (50 mg/mL) concentrations, reverses the increase in skin pigmentation induced by UVB, and also improves skin elasticity and collagen fibers [92]. Although this study did not provide direct information about GCG's effect on ROS generation or intracellular antioxidant status, its effectiveness in mitigating mitochondrial aberrations – induced by UV-mediated excessive ROS generation and participate – is obvious.

To date, the direct antioxidant activity of this compound has not been specifically observed in skin cells. Moreover, the strong antioxidant activity of GCG (50 and 100 μM) has been demonstrated in mouse hippocampal neuronal HT22 cells, where GCG treatment inhibited glutamate-induced ROS accumulation [93]. On the other hand, GCG reduced ROS levels in 3T3-L1 fibroblasts in a dose-dependent manner, while simultaneously inhibiting mitogen-activated protein kinase (MAPK) pathway activity [94]. Also, GCG suppressed NF-κB activation and reduced the lipopolysaccharide (LPS)-induced expression of pro-inflammatory cytokines interleukin 6 (IL-6) and monocyte chemoattractant protein-1(MCP-1). Moreover, at high doses (20 and 40 μg/mL) GCG demonstrated cytotoxic effect on 3T3-L1 cells. Collectively, these findings highlight the promising protective potential of GCG in the skin, particularly in mitigating UV radiation-induced oxidative stress.

A study on B16F10 melanoma cells comparing the effects of three catechins (EGCG, GCG and epicatechin-3-gallate, ECG, at the concentration of 20 μg/mL) showed an anti-melanogenic potential of these compounds [95]. Both showed a modulatory effect on melanogenesis in B16F10 cells via cyclic adenosine monophosphate (cAMP) – cAMP/cAMP-responsive element-binding protein (CREB) – microphthalmia-associated transcription factor (MITF) pathway [95]. These catechins effectively suppressed tyrosinase activity and melanin synthesis, with ECG showing the greatest potency, followed by EGCG and GCG [95]. Moreover, a molecular docking study demonstrated that GCG can bind to the melanocortin 1 receptor (MC1R) [96], a crucial regulator of melanocyte proliferation and function [97]. The same study also demonstrated downregulation of MC1R expression [96]. However aberrant melanogenesis can cause cytotoxic and mutagenic effects, stimulate glycolysis and hypoxia-inducible factor 1-alpha (HIF-1α) activation, and promote melanoma progression and resistance to immunotherapy [98]. Inhibition of melanogenesis has been suggested as a strategy to enhance the efficacy of immunotherapy, radiotherapy, and chemotherapy in advanced melanotic melanoma [98]. Considering the potential role of GCG in melanogenesis, further research is needed to evaluate its inhibitory effect, in detail, on this process.

An early study showed that the synthetic GCG analog, (+)–GCG (0.01 and 100 μM), selectively inhibits chymotrypsin-like activity in both purified 20S and 26S proteasomes from tumor cell lysates (human Jurkat T and prostate cancer cell lines, LNCaP, DU-145) [99]. While the antioxidant properties of natural GCG have been noted in non-skin cancer contexts, its impact on proteasome function, protein aggregation, and oxidative protein modifications has yet to be specifically elucidated. However, considering the potential of GCG to downregulate NF-κB activation, its possible role as a proteasome inhibitor should be evaluated in the context of skin cancers characterized by constitutive NF-κB activation [100]. By controlling NF-κB signaling, the ubiquitin-proteasome system serves as a key mediator of stress response pathways in the skin [100].

Despite its potential, research on GCG's role in skin cancer remains limited, especially concerning SCC and BCC. On the other hand, GCG has been tested for proliferation of the cancer cell lines (breast, central nervous system, colon and lung) [101]. GCG, presented a significant anti-proliferative effect on mentioned cell lines (at 50 μM concentration for breast, colon and lung while at 100 μM concentration for central nervous system) [101]. A water extract of *Camellia ptilophylla* rich in GCG (≥98 %) demonstrated significant tumor growth inhibition in mice bearing HCT116 colon cancer [102]. Moreover, same study demonstrated HCT116 cell apoptosis through ROS generation mediated mitochondrial dysfunction and PI3K/Akt inactivation [102]. Another study reported a significant reduction in the growth, migration, and invasion of 4T1 murine breast cancer cells following treatment with *Zhangping narcissus* tea cake extract, which contains a high level of GCG [103]. While not directly linked to *Camellia sinensis* or pure GCG, the findings highlight GCG's therapeutic potential, especially in cancer metabolism. Therefore, a detailed investigation of cell death pathways, ROS dynamics, associated lipid metabolism, and inflammatory responses – specifically in the context of skin cancer – is recommended.

#### 4.1.3 (−)-catechin-3-O-gallate (CG; PubChem CID: 6419835)

Another polyphenolic compound, the gallate ester (*−*)-catechin-3-*O*-gallate (CG), has emerged as a promising agent for alleviating or preventing skin damage. Notably, data on this catechin – particularly in the context of skin applications – remain limited compared to other catechins. This may be attributed to demonstrated highly effective free-radical scavenging activity of the other two catechins, EGCG and (*−*)-epicatechin gallate (ECG), likely due to the presence of hydroxyl groups in their gallate moieties [104]. Together with this, CG physical stability under UV exposure is notably high. Structural analyses report that it is UVB-insensitive, maintaining 90.7 %–99.6 % of structural integrity under 6h UVB radiation [105].

On the other hand, an earlier study demonstrated that both synthetic and natural enantiomers of CG inhibited the caspase-like activity of the 26S proteasome in leukemia Jurkat T cell extracts [106]. A study reported that CG (50 μM) exhibits inhibitory activity against sphingomyelinase – albeit weaker than ECG – in rats at physiological plasma concentrations [107]. Importantly, sphingomyelinases – which are involved in the generation of ceramides, critical bioactive lipids associated with apoptosis, oxidative stress, and inflammation – appear to be important targets for developing new therapeutic applications in cancer settings [108]. However, there is very limited research exploring the molecular mechanisms of CG action, directly. Considering above, detailed functional studies are needed to demonstrate its efficacy in skin applications, particularly regarding its therapeutic potential in skin cancer.

Studies in other cancer types support the importance of investigating the role of CG in skin cancer. A recent review on breast cancer cells reported that CG specifically interferes with the NF-κB pathway, by reducing p65 expression and activity [109]. Additionally, the CG-mediated intracellular molecular response involves the regulation of cell death (JAK/STAT pathway) and ATP-binding cassette transporters, as well as alterations in oncogene activity (p53) and the regulation of DNA repair mechanisms (nucleotide excision repair, NER; ataxia telangiectasia mutated protein, ATM) [109].

A comprehensive research study using both prostate and breast cancer cell lines demonstrated that CG, isolated from *Acacia hydaspica*, effectively suppressed cell survival and colony formation as evidenced by chromatin condensation, formation of apoptotic bodies, and cell shrinkage (100μM of CG for PC-3 cells and 50 μM of CG for MDA-MB-231 cells) [110]. The changes observed in the prostate cancer cell line were together with the downregulation of anti-apoptotic proteins Bcl-2, Bcl-xL, and survivin expression and, the suppression of related signaling pathways, including NFκB (p65 phosphorylation), extracellular signal-regulated kinases 1 and 2 (ERK1/2), AKT, and JAK/STAT [110]. In breast cancer cell lines, the reduction in cell growth was accompanied with decreased expression of survivin, B-cell lymphoma-extra large (Bcl-xL), casein kinase II subunit alpha (CK2α), and X-linked inhibitor of apoptosis (XIAP), along with the suppression of NFκB, JAK/STAT, and PI3K signaling pathways [110].

Importantly, alongside metabolic differences arising from the genetic profiles of metastatic cancer cells, such as variations in oxidative phosphorylation, glycolysis, and glutaminolysis pathways, similarities have also been identified in the metastatic metabolomes of aggressive prostate, breast, and melanoma cells [111]. For instance, metabolites like creatine and phosphocreatine are commonly found across these cancers, while uridine diphosphate N-acetylglucosamine (UDP-GlcNAc) is shared between melanoma and prostate cancer cell lines [111]. Accordingly, taking into account both the differences and the underlying similarities, CG can be regarded as a promising candidate for further in-depth investigation as a therapeutic agent against skin cancer, both melanoma and NMSC, on both early and advanced stages of development. However, it is absolutely essential that both CG, discussed herein, and other catechins are thoroughly evaluated for potential side effects and drug interactions, considering their highly bioactive nature. In fact, it has been shown that the drug-metabolizing enzyme cytochrome P450 2C (CYP2C) is strongly inhibited by CG (7.60 μM) [112].

### 4.2 Procyanidin C1 (PCC1, PubChem CID: 169853)

Procyanidin C1 (PCC1) is a B-type proanthocyanidin, composed of three epicatechin units, classified as a flavonoid and found in grapes [113,114]. Recent data highlight the therapeutic importance of PCC1, resulting from its phenolic structure, and its senolytic properties that alter the microbiome, as well as its anti-inflammatory and redox-regulating effects [115,116]. Furthermore, its tendency to localize at the interface of biological membranes and spontaneously form higher-order oligomers suggests that its bioactive properties may be related to its membranotropic effects, potentially opening new avenues for future therapeutic interventions [117].

The antioxidant activity of PCC1 – including free radical scavenging, metal ion chelating, and reduction of hydroperoxide levels – is determined by its degree of polymerization [116]. As a member of the procyanidin family, PCC1 typically acts as an antioxidant, reducing ROS production and alleviating oxidative stress and increasing the viability of mammalian cells, as observed for example in pancreatic β-cells exposed to H_2_O_2_ [118]. Furthermore, a study on senescent PSC27 cells (mice) showed that PCC1 (100 μM) increased ROS levels, suggesting that it may induce mitochondrial dysfunction and regulate apoptosis, especially in senescent cells [116]. These results indicate the biological activity, efficacy, and mechanism of action of PCC1 influenced by factors such as cell type and the surrounding cellular microenvironment.

Moreover, a recent study using HaCaT cells exposed to UVB radiation showed that PCC1 reduces ROS levels and, consequently, their interactions with cellular macromolecules –leading to a decrease in lipid peroxides and an increase in collagen expression [119]. It was also shown that PCC1, piperitoside and mulberrofuran E, reduces the expression of MMP1, tumor necrosis factor alpha (TNF-α) and inducible nitric oxide synthase (iNOS), while increasing the expression of type I collagen (COL1A1), β-catenin (CTNNB1) and SOD1 [119]. These findings highlight the protective potential of these compounds in preventing metabolic destruction in skin cells under the influence of UVB radiation.

Although the data on the molecular activity of this compound on skin cells are limited, studies using other cell types offer important metabolic insights. PCC1 (5 and 10 μM) has been also shown to exert neuroprotective effects on HT22 cells by significantly reducing ROS generation and protein carbonylation, increasing Nrf2 activity and heme oxidase-1 (HO-1) expression, inhibiting the phosphorylation of MAPKs such as ERK1/2 and p38 – but not c-Jun N-terminal kinase (JNK) – and preventing glutamate-induced apoptosis [120]. Another study also highlights the neuroprotective effects of PCC1 (25 μM) in PC12 cells by regulating the efficiency of the Nrf2 pathway and increasing cell survival [121]. Moreover, similar effects of PCC1 (2.5 μM and 5 μM) have also been reported in a zebrafish model of Parkinson's disease [122]. Furthermore, a study on LPS-stimulated RAW 264.7 macrophages with ethanolic extract of *Rosa laevigata* Michx. (FR) fruits containing PCC1 showed a significant anti-inflammatory effect by inhibiting nitric oxide production and MAPK/NF-κB signaling after activation of adenosine monophosphate-activated protein kinase (AMPK) [123].

The ability of PCC1 to modulate intracellular redox balance, inflammatory status, and associated signaling pathways highlights its significant therapeutic potential for skin applications. As mentioned above UV radiation causes constant exposure of skin cells to oxidative stress and inflammation. Considering the crucial role of lipids and their metabolites in inflammation and cell survival, especially in the context of impaired redox balance [[124], [125], [126]] caused by UV exposure, further studies are necessary to explain how PCC1 influences the dynamics of lipid metabolism through its antioxidant and anti-inflammatory effects under the condition of UV-mediated oxidative microenvironment.

The multifaceted metabolic actions of PCC1 are revealed both *in vitro* and *in vivo*. By binding to EGFR and inhibiting its phosphorylation, suppressing multiple signaling cascades (ERK/MAPK and AKT/mTOR), and ultimately disrupting TGF-β/SMAD signaling, PCC1 (12.5 μM and 25 μM) has been found to exhibit antifibrotic activity in male C57BL/6J mice [127]. In contrast, fibrosis is increasingly recognized as a contributing factor in the development of various cancers including breast, pancreatic, lung cancers as well as melanoma, making it a key factor in drug adaptation and therapeutic resistance, and thereby crucial for tailoring cancer therapy [128]. Therefore, a thorough assessment of the impact of PCC1 on wound healing, chronic fibrosis, and neoplastic pathways is crucial to understand its potential in melanoma development as well as skin carcinogenesis in general. This is supported by the results indicating that PCC1 inhibits melanoma cell growth via activation of 67LR–protein kinase A (PKA)/protein phosphatase 2A (PP2A)/protein phosphatase inhibitor-1 of 17 kDa (CPI17)/myosin regulatory light chain (MRLC) C-kinase signaling [129]. These results underscore the antitumor potential of PCC1, further supported by rodent models showing that PCC1 (100 μM) removes senescent cells in the microenvironment of treatment-damaged tumors and enhances chemotherapy efficacy when PCC1 used in combination with the chemotherapy [116]. Furthermore, a study evaluating the association between aging-induced changes in the immune microenvironment and melanoma development, as well as lung metastasis, in male mice showed that PCC1 (20 mg/kg; i.p.) reversed the aging-dependent reduction in the tumor-killing capacity of CD8^+^ T cells via the IL-17-expressing γδT cell-neutrophil-CD8^+^ T cell axis [130].

Although there is no direct data in the literature yet regarding the involvement of PCC1 in the development-treatment of NMSC, its demonstrated efficacy against other types of cancers highlights its promising therapeutic potential. PCC1 (10 μM) has been shown to inhibit tumor growth and metastasis in colon cancer by inhibiting miR-501-3p and preserving HIGD1A (hypoxia-inducible gene domain family member 1A) expression [131]. In breast cancer cell lines (MDA-MB-231 and MCF-7), PCC1 showed antiproliferative activity by inducing DNA damage, increasing the expression of BCL2 associated X (BAX), caspase-3 and caspase-9, downregulating the expression of Bcl-2 and ultimately promoting apoptosis [132].

PCC1 (20 mg/kg; 2 days) inhibits connective tissue growth factor (CTGF) signaling, which enhances the efficacy of 5-fluorouracil (5-FU) chemotherapy, along with ion channel protein PIEZO1 depletion by short hairpin RNAs (shRNA), ultimately suppressing peritoneal metastasis of gastric cancer cells [133]. Another study demonstrated the anticancer effects of a combination treatment with malvidin-3-*O*-(6-*O*-coumaroyl)-glucoside-5-*O*-glucoside (M35GC) and PCC1 in the MKN-28 gastric cancer cell line [134]. This treatment induced apoptosis by reducing the Bcl-2/Bax ratio. It also causes G0/G1 phase cell cycle arrest by downregulating CDK4 (cyclin-dependent kinase 4) protein levels, and it decreases both glucose consumption and lactate production. Moreover, an interesting study demonstrated the anti-tumor activity of PCC1 (20 mg/kg; i.p.) in a subcutaneous tumor mouse model [135]. This model was created by injecting cancer-associated fibroblasts and gastric adenocarcinoma MKN-45 cells into NSG mice. In addition, *Cinnamomi cortex* extract, which primarily contains PCC1, showed significant inhibitory activity against TGF-β-induced epithelial-to-mesenchymal transition (EMT) [136]. This effect was observed in A549 lung adenocarcinoma cells.

**Fig. 4:**
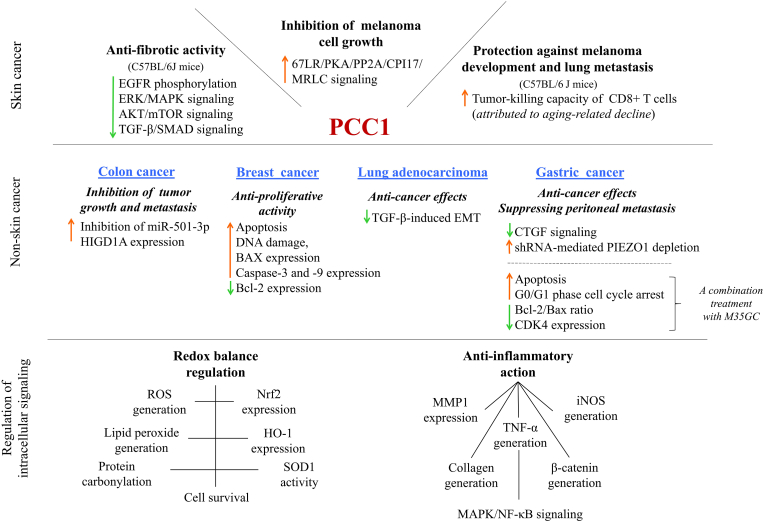
Procyanidin C1 (PCC1) interferes with intracellular signaling by modulating both redox and inflammatory pathways. Its observed anti-fibrotic effects and inhibition of melanoma cell growth highlight its therapeutic potential against skin cancer. Furthermore, its anti-cancer effects on colon, breast, lung, and gastric cancer cell lines strongly support this potential. [**67LR**, 67-kDa laminin receptor; **Akt**, protein kinase B; **BAX**, BCL2 associated X; **Bcl-2**, B-cell lymphoma 2; **CD8^+^ T cells**, cytotoxic T lymphocytes; **CDK4**, cyclin-dependent kinase 4; **CPI17**, protein phosphatase inhibitor-1 of 17 kDa; **CTGF**, connective tissue growth factor; **EGFR**, epidermal growth factor receptor; **EMT**, epithelial-to-mesenchymal transition; **ERK**, extracellular signal-regulated kinase; **HIGD1A**, hypoxia-inducible gene domain family member 1A; **HO-1**, heme oxidase-1; **iNOS**, inducible nitric oxide synthase; **M35GC**, malvidin-3-*O*-(6-*O*-coumaroyl)-glucoside-5-*O*-glucoside; **MAPK**, mitogen-activated protein kinase; **miRNA**, micro RNAs; **MMP1**, matrix metalloproteinase 1; **MRLC**, myosin regulatory light chain; **mTOR**, mammalian target of rapamycin; **NF-κB**, nuclear factor kappa B; **Nrf2**, nuclear factor erythroid 2-related factor 2; **PKA**, protein kinase A; **PP2A**, protein phosphatase 2A; **ROS**, reactive oxygen species; **shRNA**, short hairpin RNAs; **SOD1**, superoxide dismutase 1; **TGF-β**, transforming growth factor-beta; **TNF-α**, tumor necrosis factor-α].

The metabolic effects of PCC1, including its anticancer activity observed in *in vitro* studies, suggest its protective and therapeutic potential against cancers, including skin cancers (Fig. 4). Therefore, both *in vivo* studies and clinical trials are necessary to confirm the *in vitro* findings related to this compound.

### 4.3 Piperitoside (PubChem CID: 131752173)

Another promising compound that is both a glycoside and a flavonoid is piperitoside [137]. It is known, however, that flavonoid glycosides are widespread in plants and, by acting as phytoalexins against biotic stress, may exert beneficial health effects in humans [138]. Glycosylation significantly influences the structural complexity, water solubility, stability, and ultimately the bioactivity of flavonoids [138,139]. They exhibit significant antioxidant, immunomodulatory, and anticancer effects [140]. A recent literature review highlights the bioactive properties of plant-delivered bioactive compounds – including piperitoside, found in *Mentha piperita* – which has shown beneficial effects in skin irritation, redox imbalance, and inflammation [141]. However, current literature provides only limited information on the mechanisms underlying its biological effects in skin cells and other tissues.

As mentioned above in section 3.2 on PCC1, analysis of HaCaT cells exposed to UVB radiation showed that piperitoside exhibits significant multi-targeted antioxidant and anti-inflammatory activities [119]. Furthermore, following UVB exposure, piperitoside inhibits ROS production, lipid peroxidation, and the expression of MMP1, TNF-α, and iNOS, while increasing the expression of SOD1, COL1A1, and CTNNB1 [119]. Although there is no direct data in the scientific literature demonstrating the influence of piperitoside on other molecular activities, intracellular signaling, or related cellular responses, the present findings suggest its potential for maintaining healthy skin and/or the therapeutic potential against skin cancer. However, these findings highlight the need for a more detailed investigation into the metabolic actions of PCC1.

**Fig. 5:**
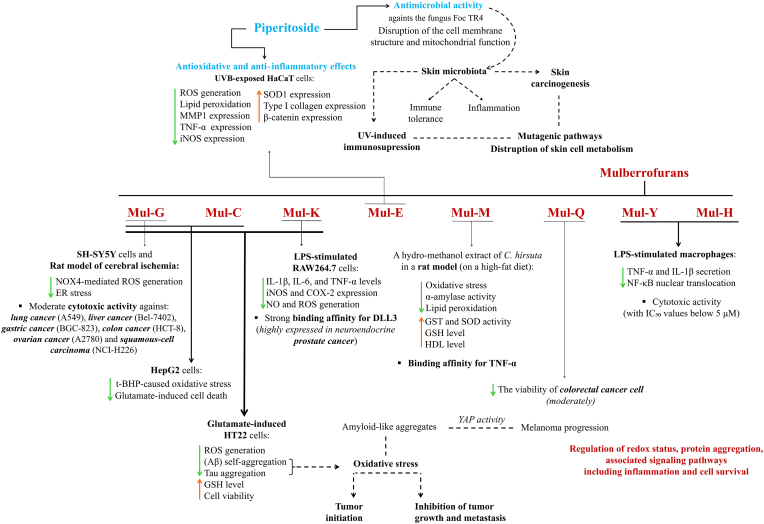
Piperitoside and mulberrofuran G, C, K, E, M, Q, Y, and M (abbreviated as Mul-G, Mul-C, Mul-K, Mul-E, Mul-M, Mul-Q, Mul-Y, and Mul-M) exhibit therapeutic potential for skin cancer prevention and therapy. Piperitoside exhibits significant antioxidant, anti-inflammatory, and antimicrobial activities, which may modulate the skin microbiota – an important factor dynamically associated with UV-induced immunosuppression and skin carcinogenesis. Mulberrofuran G, C, K, E, M, Q, and Y have been shown to modulate intracellular redox status, protein aggregation, and related molecular signaling pathways involving inflammation and cell survival – key processes in the initiation and progression of skin cancer in which oxidative stress and protein aggregation play critical roles. The cytotoxic activity of these mulberrofurans has been demonstrated across multiple cancer cell lines. [**Aβ**, β-amyloid; **COX-2**, cyclooxygenase-2; **DLL3**, delta-like ligand 3; **ER**, endoplasmic reticulum; **Foc TR4**, fusarium oxysporum f. sp. cubense tropical race 4; **GSH**, glutathione; **GST**, glutathione S-transferase; **HDL**, high-density lipoproteins; **IL-1β**, interleukin-1 beta; **IL-6**, interleukin 6; **iNOS**, inducible nitric oxide synthase; **LPS**, lipopolysaccharide; **MMP1**, matrix metalloproteinase; **NF-κB**, nuclear factor kappa B; **NO**, nitric oxide; **NOX4**, NADPH oxidase 4; **ROS**, reactive oxygen species; **SOD1**, superoxide dismutase 1; **t-BHP**, *tert*-butyl hydroperoxide; **TNF-α**, tumor necrosis factor alpha; **YAP**, yes-associated protein.

Furthermore, a study using an extract of *Streptomyces hygroscopicus* subsp. *hygroscopicus* 5-4 containing piperitoside and six other major differential metabolites demonstrated potent antifungal activity [142]. The extract was found to disrupt the cell membrane structure and mitochondrial function of the fungus *Fusarium oxysporum* f. sp. *cubense* tropical race 4 (Foc TR4) [142]. It is known that the skin microbiota influences immune tolerance and inflammation through its metabolites interacting with the immune system [143]. Disturbances in the composition and diversity of the skin microbiota have been linked to various skin diseases, including skin carcinogenesis [143]. This represents a new and important area of interest with significant potential for research into modifications of the skin's metabolic response. Current literature highlights the relationship between changes in the skin microbiota, UV-induced immunosuppression, and the involvement of the skin microbiota in the activation of mutagenic pathways [144]. This also indicates the need for further research on the role of the skin microbiome in cancerous skin [144]. Therefore, the potential antimicrobial effects of piperitoside, as mentioned earlier, confirms its potential as a candidate for further research in the prevention and/or pharmacotherapy of skin cancer (Fig. 5).

### 4.4 Mulberrofurans

Another group of biologically active polyphenols are mulberrofurans – benzofuran-type polyphenols isolated from *Morus* species, which exhibit diverse biological activities, including antioxidant and anti-inflammatory effects [[145], [146], [147]]. Mulberrofuran G (0.2, 1 and 5 mg/kg (PubChem CID: 9959532) was shown to reduce NOX4-mediated ROS generation and endoplasmic reticulum stress in both *in vitro* (SH-SY5Y cells) and *in vivo* (middle cerebral artery occlusion/reperfusion-induced ischemic rats) models of cerebral ischemia [146]. Both mulberrofuran G and mulberrofuran C (PubChem CID: 157143) demonstrated hepatoprotective and neuroprotective activities, respectively, in *tert*-butyl hydroperoxide (t-BHP)-induced oxidative stress in HepG2 cells and glutamate-induced cell death in HT22 cells [148]. On the other hand, the protective effects of four compounds (mulberrofuran C, mulberrofuran K (PubChem CID: 495296), mulberrofuran G, and isomulberrofuran G) counteracting oxidative stress and neuroinflammation have been evaluated in a glutamate-induced HT22 cell model, showing increased GSH levels and decreased ROS production [149]. Moreover, above study revealed that both compounds significantly restored the viability of cells treated with glutamate and reduced β-amyloid (Aβ) self-aggregation as well as tau aggregation [149]. These findings clearly indicate that mulberrofurans can modulate cellular response to oxidative stress and associated protein aggregation [150]. Redox-regulatory activity of mulberrofurans, associated with changes in cellular protein misfolding and aggregation status, suggests their significant therapeutic potential in counteracting skin cancer, particularly melanoma. Oxidative stress is considered a paradoxical factor, as it can both promote tumor initiation and inhibit vertical growth and metastasis [151]. In addition to protein misfolding, p53 also undergoes biomolecular condensates and aggregates analogous to other protein-based amyloids, significantly influencing cancer progression through loss-of-function, dominant-negative, and gain-of-function mechanisms [152]. Importantly, similar to the aggregates observed in Alzheimer's disease, amyloid-like aggregates have also been detected in human melanoma biopsies, where they induce the activity of yes-associated protein (YAP), a factor involved in the increased transcriptional activity associated with melanoma progression [153].

The anti-inflammatory effect of mulberrofuran K was also demonstrated in LPS-stimulated RAW264.7 cells by reducing the levels of IL-1β, IL-6, and TNF-α, as well as decreased expression of iNOS and COX-2, accompanied by decreasing NO and ROS production [154]. A study evaluating the hypoglycemic effect of a hydro-methanol extract of *C. hirsuta* (500 mg/kg) in a rat model highlighted the antidiabetic effects of mulberrofuran M (PubChem CID: 21594897) and quercetin-3-(6″-caffeoylsophoroside), attributed to their ability to reduce oxidative stress and inhibit α-amylase [155]. Molecular docking analysis revealed that mulberrofuran M exhibits a binding affinity for TNF-α [155]. Furthermore, *in vivo* results demonstrated a reduction in lipid peroxidation, accompanied by increased levels of high-density lipoproteins (HDL), enhanced activity of glutathione S-transferase (GST) and SOD, and elevated level of GSH [155]. Although these naturally occurring compounds are being studied for their biological activity, their effects on skin cells – particularly in the context of skin cancer – remain largely unknown. Thus, their demonstrated activity in both healthy and cancerous cells supports their potential as promising agents in skin cancer treatment and paves the way for future research.

Although data on normal skin and cancer cells (both cutaneous and from other tissues) remain limited, the available evidence underscores the therapeutic potential of mulberrofurans. As mentioned for piperitoside and procyanidin C1, mulberrofuran E (PubChem CID: 100930829) has also been shown to counteract UVB-induced prooxidant and proinflammatory cellular responses in HaCaT cells [119]. Similar to the other two compounds, mulberrofuran E significantly reduces ROS and lipid peroxide levels, as well as the expression of MMP1, TNF-α, and iNOS, while increasing the expression of collagen and SOD1 [119]. These results highlight the effects of mulberrofuran E on redox balance and related protein signaling, as well as its ability to protect the skin from UV-induced damage. However, these findings require confirmation through further detailed studies.

Importantly, mulberrofuran K exhibited strong binding affinity for delta-like ligand 3 (DLL3), which is highly expressed in neuroendocrine prostate cancer, as well as in most tumors with features of high-grade or small cell neuroendocrine cancer, and also inhibited the Notch signaling pathway [156,157]. Furthermore, mulberrofuran Q (PubChem CID: 5319933) has been shown to moderately reduce the viability of colorectal cancer cell lines, suggesting its potential therapeutic efficacy (0.1, 1, 10 and 100 μM) [158].

In contrast, mulberrofurans Y (PubChem CID: 10621441) and H (PubChem CID: 20056162) have demonstrated cytotoxic activity with IC_50_ values below 5 μM, along with a reduction in TNF-α and IL-1β secretion and inhibition of NF-κB nuclear translocation in LPS-stimulated macrophages [159,160]. However, mulberrofuran G also exhibited moderate cytotoxic activity against six human cancer cell lines: lung cancer (A549), liver cancer (Bel-7402), gastric cancer (BGC-823), colon cancer (HCT-8), ovarian cancer (A2780) [161], as well as squamous-cell carcinoma (NCI–H226) [162]. Although these results do not directly demonstrate the efficacy of these mulberrofurans in the treatment of skin cancers, they clearly show their influence the metabolism and survival of cancer cells, particularly in the other cancer types mentioned above (Fig. 5). Therefore, studying the effects of mulberrofurans on skin cancer cells may yield results that are important for pharmacotherapy.

## 5 Implications of phenolic compounds for dietary and therapeutic applications

Phenolic (natural) compounds are of growing interest as both dietary constituents and therapeutic agents, owing to their dose-dependent ability to modulate redox signaling, thereby functioning as either antioxidants or prooxidants. However, significant issues remain regarding both daily dietary intake and the potential translation of these findings into effective therapeutic strategies. Here, a detailed analysis of the metabolism, pharmacokinetics, and dosing of these highly bioactive compounds, along with their short- and long-term effects, is essential.

### 5.1 Dietary consumption

Regardless of their specific therapeutic effects, recent reports highlight that dietary flavonoid intake is linked to a reduced risk of major chronic diseases and overall mortality [163]. Literature suggests that dietary polyphenols exhibit strong antioxidant, anti-inflammatory, anti-microbial, and anti-glycation properties, which may help counteract diseases such as Alzheimer's disease, diabetes, coronary heart disease, and cancer [164]. Nonetheless, important challenges persist concerning daily dietary intake of them. For instance, pharmacokinetic data and reported cases of hepatotoxicity suggest a potential risk associated with the consumption of green tea and its preparations. This concern arises from the internal dose of catechins, given their highly bioactive nature [165]. Moreover, clinical trials have revealed considerable inter-individual variability in response to polyphenol intake [166].

Additionally, dose equivalence of above compounds needs to be thoroughly optimized in conjunction with improvements in bioavailability, thorough evaluation of dose equivalence across species (regarding *in vivo* animal studies and clinical trials) alongside stringent clinical validation [164]. In the context of normal dietary consumption – therapeutic applications as well – one of the most critical factors for the efficacy and safe use of phenolics is the dosage and duration of administration. Phenolic compounds may exhibit pro-oxidant activity due to their redox properties and their tendency to undergo self-oxidation at higher concentrations [167]. There are several case reports of acute liver injury, including hepatocellular necrosis and cholestasis, linked to green tea extracts containing high concentrations of EGCG, particularly more than 400 mg per day [168]. Also, EGCG administration has been associated with reports of adverse effects, including hepatotoxicity [169]. On the other hand, clinical trials indicate that intake of EGCG doses of 800 mg/day or higher, taken as a dietary supplement, leads to a statistically significant increase in serum transaminase levels compared to control subjects [170]. Regarding regular dietary supplements, the variability in hepatotoxic responses has been attributed to dose- and cell/organism-specific effects, and eventually, its act as antioxidant/pro-oxidant [171], as mentioned above. Thus, further research is needed to analyze the effects of administered dosage, epigenetic factors, and the duration of treatment/dietary supplementation. Also, given the lack of trial data on the long-term toxicity of both EGCG and other phenolic compounds, further clinical studies are necessary.

Compared to the other compounds discussed in this paper, the metabolism of EGCG is better characterized. Literature indicates that EGCG metabolism and bioavailability significantly influence its effects on lipid metabolism and inflammation, highlighting its potential for skin protection. It is mainly absorbed in the intestine. Before absorption, large polyphenolic molecules are broken down by the gut microbiota into smaller bioactive forms that boost EGCG's systemic activity [172]. In addition, its detectable plasma concentrations in fasting individuals after oral intake indicate good bioavailability [173]. Following oral administration, EGCG is predominantly eliminated as metabolites via bile and urine, with only trace amounts of the unchanged compound detectable in urinary excretion [174]. Therefore, its oral administration represents a significant advantage, particularly in the context of dietary consumption.

Importantly, the data obtained so far highlight EGCG safety and lack of harmful side effects, for the doses between 800 and 1600 mg (using once-daily and twice-daily regimens) [172]. Only mild side effects have been reported, such as stomach discomfort, abdominal pain, nausea, and headaches [172]. Evidence suggests that green tea, especially EGCG, may contribute through dietary supplementation to the prevention and management of metabolic syndrome and its associated age-related disorders [175].

### 5.2 Therapeutic use

Beyond dietary consumption, further research is necessary to fully understand the potential of phenolic (natural) compounds as therapeutic agents. Given their potent antioxidant properties and radical-scavenging capacity, the discrepancies observed between preclinical and clinical studies on antioxidant therapies warrant a detailed examination from a broader pharmacological perspective. Despite the promising effects of phenolics reported in this and other studies, a comprehensive evaluation of their bioavailability, stability, pharmacokinetics, and dose equivalence is still needed, with particular emphasis on reinforcing cross-species dose comparison studies, as suggested before [164].

Studies underline the low efficiency of transdermal delivery and the photodegradation of EGCG (having high molecular weight) in topical formulations limit its clinical application for external use [176]. Together with that, topical application of EGCG (50 mg/kg) has been shown to effectively deliver sustained levels of EGCG to plasma and tissues [177]. It has been also shown that EGCG bioavailability and stability can be improved by the encapsulating of EGCG (400 μg/g) in emulsified oil droplets [178]. Furthermore, given the advantages of topical drug delivery – such as avoiding gastrointestinal irritation, bypassing hepatic first-pass metabolism, and minimizing side effects [179] – optimizing topical application strategies for phenolic compounds is crucial for their effective therapeutic use on the skin, including as potential adjuvant treatments in cancer therapy.

Controlled *in vitro* studies, differing from natural complex biological systems, and the species-related variations of *in vivo* models, such as in drug metabolism, all directly influence the practical translation of the observed efficacy of phenolics in preclinical studies. Phenolic compounds are ingested and metabolized by various mechanisms, including glucuronidation and methylation, which can alter their biological activity [180]. Notably, they can also be catabolized by colonic microflora, markedly affecting their absorption across the gut barrier [180]. Their metabolism (regarding the compounds discussed herein and others) is not well represented. Particularly regarding the effects of microbial metabolites [181], further research – especially systematic studies utilizing omics approaches – is needed to elucidate the metabolism of phenolic compounds in the context of polyphenol-microbiota-host interactions [164]. Moreover, the regulation of UV-related immunosuppression in the context of the relationship between the human microbiota and skin cancer is still unclear and remains a subject of interest [144]. Given the individual variations observed in cancer biology as well as the differential responses of cancer cells to phenolic compounds reported in clinical studies, this is critical for developing personalized treatment approaches in cancer therapies.

The natural occurrence of EGCG and its low toxicity, as explained above, make it a promising candidate for anticancer therapy, with potential to augment conventional treatments through additive or synergistic effects [172]. Beyond that, EGCG safety, metabolism and bioavailability should also be evaluated for its efficacy in skin cancer cells, as these cells differ from healthy ones in terms of metabolic activity, genetic differences, and intracellular signaling dynamics. On the other hand, data on the metabolism of the other two catechins remain limited. However, studies have demonstrated that green tea catechin stereochemistry can affect their transcellular transport and metabolism [182]. And, due to their extensive metabolism (via phase II processes) as well as catabolism by colonic microbes, their pharmacokinetics can be complex and variable [183].

Which is why there is a need for a detailed investigation into the metabolism of procyanidin C1, piperitoside, and mulberrofurans. Proanthocyanidins from different sources have varying structures, and even minor structural differences are known to influence their regulation of blood glucose [184]. The crucial role of colon microbiota has been also underlined for proanthocyanidins [185]. Most proanthocyanidins pass through the colon intact and are broken down into phenylvalerolactones and phenolic acids by the colonic microbiota [185]. Thus, research on the changes in colonic microbiota is important for determining the effectiveness of procyanidin C1 and other phenolic compounds on skin cancer, as it directly influences their bioavailability and pharmacokinetics. Importantly, recent research also underscores the significance of the gut-skin axis in connecting microbiota dysbiosis with skin inflammation [186]. Changes in both the skin and gut microbiome need to be evaluated in detail for the protective effects and efficiency of phenolic compounds, particularly in skin cancer, which is characterized by altered cell metabolism.

Together with that, a study exploring the intestinal first-pass metabolism of Mulberrofuran Y and G using the Transwell™ system with Caco-2 cells showed that, Y was largely stable, whereas G was almost completely metabolized [187]. Finally, considering the limited data on their metabolism, it is recommended that the metabolism of these compounds should be investigated in detail – both in healthy and skin cancer cells, considering the effects of the skin and gut microbiota as well – particularly to ensure that the activities observed *in vitro* can be translated into practical, real-world applications.

## 6 Conclusion

**Fig. 6:**
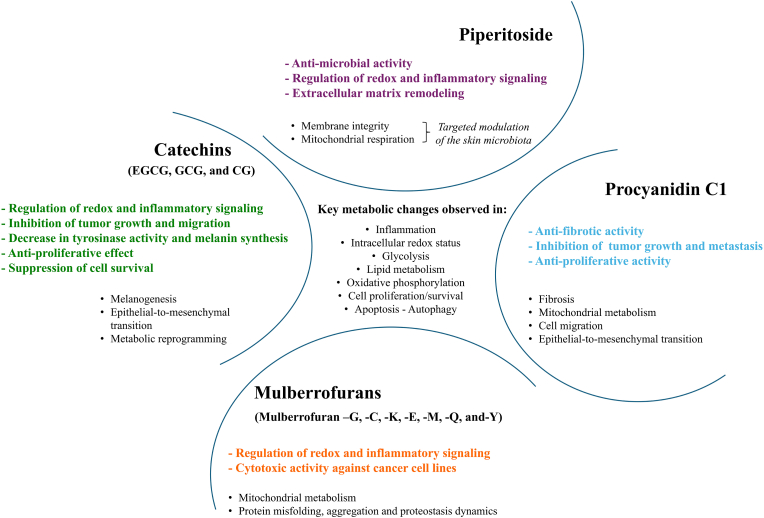
This comparison illustrates the metabolic changes (both molecular and cellular) induced by catechins, piperitoside, procyanidin C1, and mulberrofurans. The centrally indicated changes represent common molecular effects shared by all compounds, while those on the periphery are specific to each individual compound. The observed cellular changes highlight the potential of these compounds in the prevention and treatment of skin cancer.

Catechins (EGCG, GCG, and CG), procyanidin C1, piperitoside, and mulberrofuran derivatives (–G, –C, –K, –E, –M, –Q, and –Y) are promising natural phenolic compounds for the prevention and/or treatment of skin cancer. They significantly alter intracellular redox and inflammation signaling influencing lipid metabolism, and the balance between cell proliferation and survival (Fig. 6). The tumor growth-inhibitory properties of the mentioned catechins, particularly against melanoma, and the anti-fibrotic effects of procyanidin C1 also come to the forefront alongside their other biological activities. On the other hand, the role of piperitoside in the modulation of the skin microbiome, an important factor in skin carcinogenesis, as well as the cytotoxic action and regulatory effect on the protein aggregation of the mentioned mulberrofurans, justify their investigation in skin cancer research. Accordingly, comprehensive investigations of these compounds in both melanoma and NMSC are strongly recommended. The therapeutic potential of the natural compounds discussed here, along with others, holds significance for the future in addressing the complex interplay between UV exposure, skin cancer, and changing climatic and global conditions.

## AI-technologies

AI was only used to improve the clarity and quality of the English language during manuscript preparation. All content was then reviewed and revised by the authors.

## CRediT authorship contribution statement

**Sinemyiz Atalay Ekiner:** Conceptualization, Formal analysis, Investigation, Visualization, Writing – original draft. **Agnieszka Gęgotek:** Formal analysis, Visualization, Writing – review & editing. **Elżbieta Skrzydlewska:** Conceptualization, Supervision, Validation, Writing – review & editing.

## Declaration of competing interest

None. The authors have no conflict of interest to declare.

## Data Availability

No original data were generated for this review article; only literature data, as presented in the references, were used.
